# Use of human amelogenin in molecular encapsulation for the design of pH responsive microparticles

**DOI:** 10.1186/1472-6750-12-25

**Published:** 2012-05-25

**Authors:** Johan Svensson Bonde, Leif Bülow

**Affiliations:** 1 Department of Pure and Applied Biochemistry, Center for Chemistry and Chemical Engineering, Lund University, P.O. Box 124, SE-22100, Lund, Sweden

## Abstract

**Background:**

Proteins can be used in drug delivery systems to improve pharmacological properties of an active substance. Differences in pH between tissues can be utilized in order to achieve a targeted drug release at a specific location or tissue, such as a tumor. The enamel matrix protein amelogenin has a pH dependent solubility profile and self-assemble to form aggregates at neutral pH. This could make amelogenin useful in the design of pH responsive drug delivery systems.

**Results:**

In this study amelogenin was evaluated as a pH responsive component in drug delivery applications. This was achieved by testing the ability of amelogenin to entrap/release other proteins upon changes in pH, and by testing if amelogenin could confer pH responsiveness to an existing and versatile drug delivery system, such as gelatin microparticles. Amelogenin was able to encapsulate bovine serum albumin and insulin, whichwere used as model target proteins. The composite aggregates of amelogenin and target protein were formed at neutral pH and could be reversibly solubilized at weakly acidic pH. Gelatin microparticles prepared in the presence of amelogenin, showed a modulated structure in response to pH change, when studied by scanning electron microscopy, compared to particles without amelogenin. At neutral pH amelogenin induced formation of pores in the particle surface, which were not present at acidic pH, or in particles lacking amelogenin.

**Conclusions:**

The results from this study demonstrate that amelogenin can be a useful component in drug delivery systems in order to achieve a pH dependent response.

## Background

Pharmaceutical vehicles can serve several different purposes during delivery of active substances, such as extended release, increased circulation time, or protection against degradation. Lately, drug delivery systems have also been designed to facilitate targeting of the drug to a specific location in the body, for instance a cancer tumor. This could be achieved by using antibodies against antigens specific for the target tissue, or alternatively by exploring differences in chemical environment, such as differences in pH, between different tissues orcellular locations [1,2].

Protein based drug delivery systems involve use of carrier proteins to improve the pharmacological properties of the active substance. Proteins can be attractive in this context due to their generally low toxicity and high biocompatibility [3]. Examples of proteins in such systems include gelatin [4], albumin [5], casein [6] and ferritin/apoferritin [7].

Amelogenin is an extracellular matrix protein involved in formation of dental enamel during tooth development. By interacting with the apatite crystals amelogenin guides the biomineralization process that generates the highly organized enamel [8-11]. Amelogenin has very characteristic solubility properties and harbors the intrinsic ability to self-aggregate during certain physical conditions. The aggregation process is primarily affected by pH [12] and generates nanospheres with a hydrodynamic radius of approximately 20 nm [13], but larger assemblies can also be formed by subsequent aggregation of the nanospheres [14,15]. The tendency to aggregate makes amelogenin practically insoluble at physiologic pH, but the solubility is high at weakly acidic or alkaline pH [16].

The pH dependent aggregation and solubility profile of amelogenin are valuable protein properties for drug delivery applications. The aggregation behavior of amelogenin thus suggests a possible way to entrap/release other molecules, or modulate drug delivery systems to response to pH changes. The aim of this study was to assess the use of amelogenin as pH responsive component in drug delivery systems by testing its potential in pH driven entrapment of biomolecules, as well as to examine its ability to confer pH responsiveness to other frequently used drug delivery systems. In this study amelogenin encapsulation was evaluated *in vitro* using two proteins of different sizes and properties, bovine serum albumin (BSA) and insulin. In addition, the pH dependent effects of amelogenin on gelatin microparticle structure were studied by scanning electron microscopy (SEM). The biological stability of these microparticles was also examined by determining their resistance against trypsin degradation.

## Results

### Amelogenin production

Recombinant human amelogenin (rH174) was successfully produced in E. coli, using a previously described method [17]. The purified protein formed amelogenin nanospheres with a hydrodynamic radius of 17.9 ± 4.1 nm as analyzed by dynamic light scattering analysis, suggesting proper self-assembly properties.

### Molecular entrapment using amelogenin

**Figure 1:**
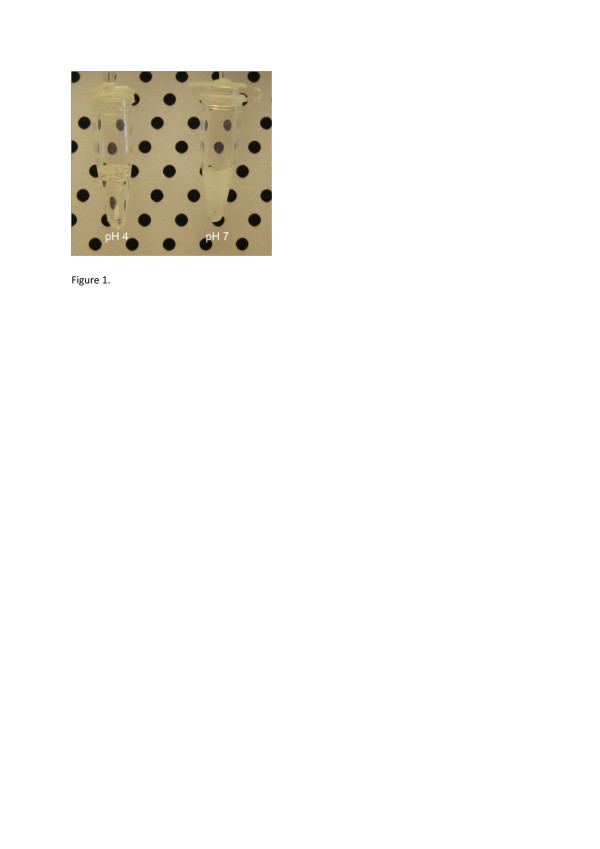
**Amelogenin samples at pH 4 (left tube) and at pH 7 (right tube). **At pH 7 amelogenin spontaneously self assembles to form insoluble aggregates.

**Figure 2:**
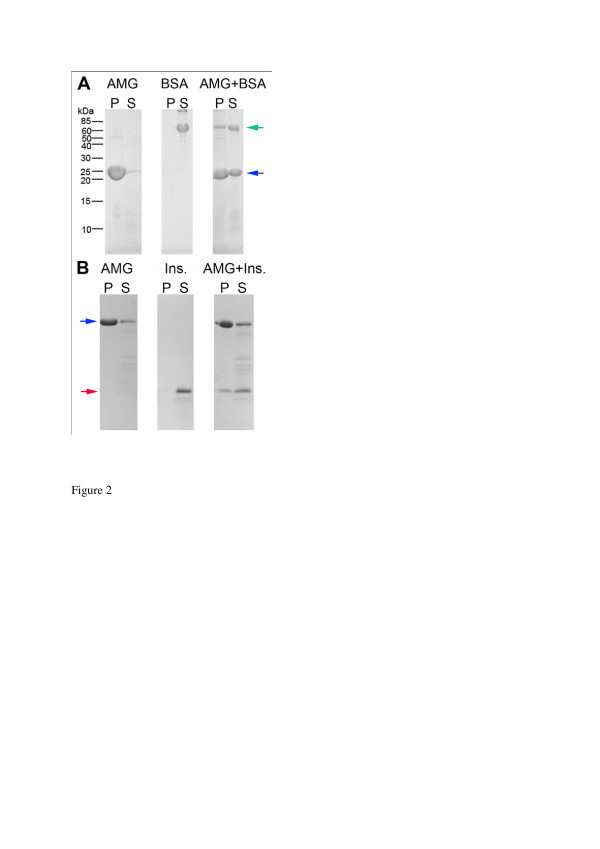
**SDS-PAGE analysis of encapsulation experiments with amelogenin (AMG) mixed with BSA (Panel A) or insulin (Ins.) (Panel B). **By changing the pH from weakly acidic to neutral the aggregation of amelogenin is induced, which entraps the target molecule. Proteins captured in the aggregates are released upon acidification will end up in the solubilized pellet sample (P). The soluble fraction (S) contains proteins not precipitated or captured by the neutralization. The three subpanels are taken from the same gel. Blue arrowsindicate amelogenin, green arrow indicates BSA, and red arrow indicates insulin.

The protein to be encapsulated (BSA or insulin) was mixed with amelogenin during slight acidic conditions, where amelogenin is soluble. By neutralizing the solution, by adding NaOH, amelogenin formed aggregates (Figure 1), which were isolated and analyzed for presence of the target protein. Encapsulation experiments with BSA showed no aggregate formation in the absence of amelogenin, and thus all BSA was found in the soluble fraction (supernatant) upon SDS-PAGE analysis (Figure 2A). However, when mixed with amelogenin in these initial encapsulation tests, a significant portion, approximately 30% of total protein located in the protein aggregates was BSA (the pellet). In these tests we used an excess of BSA, and some protein was therefore found in the soluble fraction. Entrapment of insulin with amelogenin (Figure 2B) gave similar results with 30% of the target protein located to the pellet. No insulin was found to be aggregated in the control samples.

No matter the composition, all aggregates formed at pH ~7 in this study could be solubilized at acidic pH in 0.05% HAc, indicating a reversible aggregation process, mainly affected by pH.

### Amelogenin crosslinking

**Figure 3:**
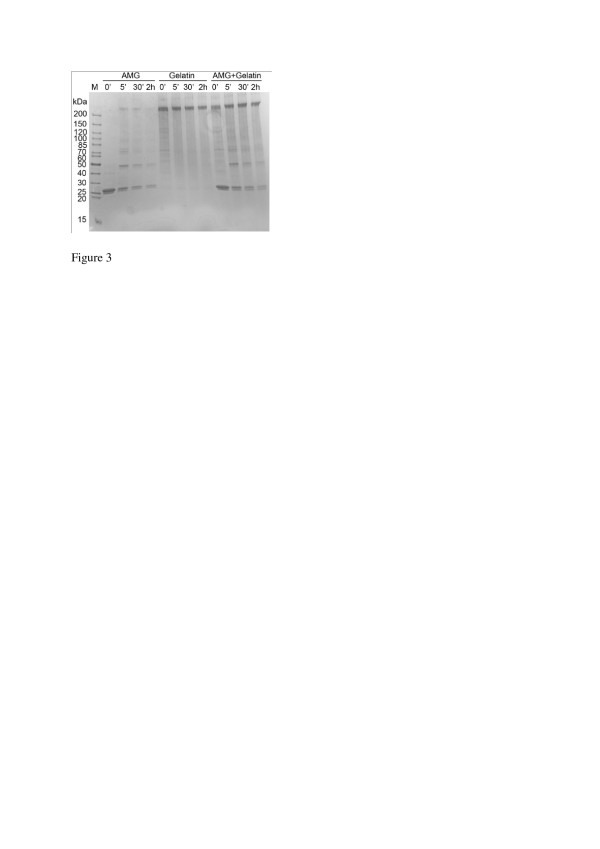
**SDS-PAGE analysis of amelogenin (AMG) and gelatin samples crosslinked with glutaraldehyde. **The samples have been crosslinked for 0, 5, 30 minutes, and 2 h. Crosslinking of amelogenin in the presence of gelatin does not alter the size of the amelogenin multimers, compared to crosslinking in the absence of gelatin. This indicates that only a minor portion of the amelogenin molecules are covalently linked with gelatin during the glutaraldehyde crosslinking reaction.

Gelatin is frequently utilized for molecular encapsulation purposes. We therefore decided to prepare mixtures of gelatin and amelogenin and also try to stabilize these composites by chemical crosslinking. Crosslinking of amelogenin with glutaraldehyde (Figure 3) resulted in rapid formation of multi- and polymeric forms, within five minutes. The same was observed for gelatin, and high molecular weight products were rapidly formed. However, when amelogenin and gelatin were mixed the subsequent addition of glutaraldehyde did not affect the amelogenin species formed, compared to samples without gelatin, indicating that very little of amelogenin was crosslinked with the gelatin at the concentrations used in our experimental setup.

### Amelogenin promotes pH responsiveness in gelatin microparticles

**Figure 4:**
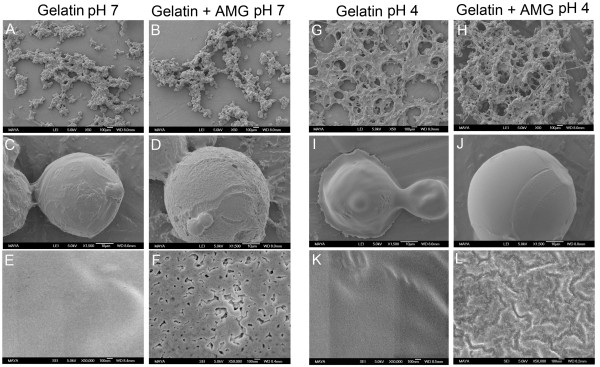
**SEM analysis of microparticles made from gelatin, with (B, D, F, H, J, L) and without (A, C, E, G, I, K) amelogenin (AMG). **The particles were incubated at pH 7 (A-F) or at pH 4 (G-L) before preparation for SEM. The presence of amelogenin affected the surface structure of the particles at pH 7 and small pores were observed in the amelogenin containing particles (panel F), but not in the control particles without amelogenin (panel E). A difference in the structure between amelogenin and control particles was also observed at pH 4 (panel G-L), but no pores seemed to form at acidic pH.

To test how pH can influence a drug delivery system composed partly of amelogenin, gelatin microparticles were created with and without amelogenin. The particles were incubated at pH 7 and pH 4 and analyzed with SEM to characterize the effect of amelogenin on the microparticle structure (Figure 4). Particle size and size distribution were not significantly affected by presence of amelogenin and in general the particles were spherical with a diameter of approximately 40–60 μm. At 1,500 x magnification the surface of the particles partly appeared heterogeneous, with both smooth and rough areas on the same particle. These different surface types could be observed on both kind of particles, but in general amelogenin containing particles exhibited a higher degree of rough structure and were less smooth. The biggest difference between the amelogenin containing particles and the control particles was observed at high magnification (50,000 x). The structure of the control gelatin particles looked smooth, even in areas that had a rough appearance at lower magnification, but in the amelogenin containing particles small pores could be observed on the particle surface. The surface pores were present in areas that looked both smooth and rough in lower magnification. The porous surfaces were only observed in the pH 7 samples when amelogenin is insoluble. The particles incubated at pH 4 showed no such pores, but the amelogenin particles had a cracked surface, in the highest magnification that was not observed in the control particles.

### Amelogenin increases microparticle stability against trypsin degradation

**Figure 5:**
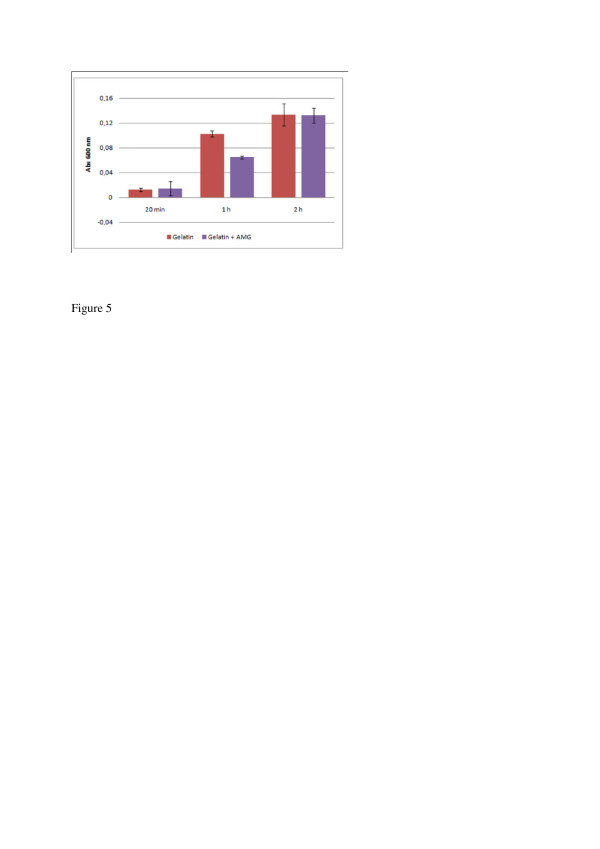
**Analysis of gelatin and gelatin/amelogenin (AMG) microparticle degradation at pH 7.0 in presence of trypsin. **The bars indicate the accumulated release of aniline blue from the particles upon proteolytic degradation, as measured by aborbance at 600 nm. The error bars represent the standard deviation based on four replicates. The amelogenin containing microparticles showed an improved resistance to trypsin degradation after 1 h incubation.

Microparticles supplemented with aniline blue were subjected to incubation with trypsin to determine the particle stability against proteolytic degradation, as a method of mimicking small intestine digestion. The accumulation of aniline blue in the sample supernatants was concomitant with degradation of the microparticles, and no aniline blue was released from the particles in the absence of trypsin. Both the amelogenin containing particles and the control particles were degraded by trypsin, but the amelogenin containing particles were significantly more resistant to the proteolysis (Figure 5). The biggest difference was observed after 1 h incubation with trypsin, where the major fraction (85%) of control particles were degraded, while 50% of the amelogenin particles were still intact. After 2 h both kinds of particles were completely degraded and no further increase in absorbance was detected.

## Discussion

The use of a drug delivery platform composed of naturally self-assembled polypeptides is often attractive due to its inherent biocompatibility and biodegradability. Amelogenin is a non-toxic and affordable protein that can easily be produced in large-scales. It has been used clinically for wound healing and dental applications for several years with no side effects reported [18-20]. Even if amelogenin often is viewed largely as an enamel associated protein, it has recently been found to be expressed in several tissues including brain, eye, cartilage and bone [21].

In order to target the release of a drug to a specific location in the body, differences in pH between tissues or cellular compartments can be utilized. For example, increased drug release at acidic pH can target a drug to a cancer tumor where the pH often is more acidic. In order to be functional the drug delivery system must be responsive to the pH change at the target site. The enamel matrix protein amelogenin has a solubility profile that is greatly affected by pH (Figure 1), which can be potentially useful in creating a pH response in drug delivery systems composed at least partly of amelogenin. In this study the use of amelogenin as a pH responsive component was assessed by testing its ability to entrap/release other proteins upon changes in pH, and by studying the effect of pH change on the structure of gelatin microparticles containing amelogenin.

The results in this study demonstrate that amelogenin can be used to entrap other proteins, in a reversible manner. BSA and insulin were found to aggregate together with amelogenin (Figure 2), suggesting formation of composite particles, containing both amelogenin and the target proteins. No BSA or insulin were found in the pellet of the control samples, indicating that the aggregates, formed together with amelogenin, were indeed composite aggregates, and not simply a mixture of two different types of aggregates. Interestingly, the presence of BSA also seemed to increase the amount of amelogenin in the soluble fraction, compared to the amelogenin control. Both amelogenin and BSA have a hydrophobic character and they may interact with each other both in solution and during aggregation, leading to increased solubility of the amelogenin part. All the amelogenin composite aggregates could be completely solubilized at acidic conditions. The reversibility of the aggregation process makes it possible to release the protein entrapped with amelogenin, following a decrease in pH. Most proteins were susceptible for entrapment in an amelogenin matrix and the amelogenin particles could carry substantial amounts of an entrapped target protein, up to 30%. Out of the proteins tested, only lysozyme encapsulation was not achieved in this study, probably due to strong protein-protein intermolecular interactions affecting amelogenin aggregation. As opposed to BSA (pI 4.8) and insulin (pI 5.3) lysozyme has an isolectric point (pI 10.7) in the far alkaline range. Although hydrophobic interactions are essential for the initial amelogenin nanosphere formation, the charged and hydrophilic C- terminus has a pronounced effect on the amelogenin aggregation. At neutral pH, lysozyme is positively charged and can thereby perturb and interfere with the native intermolecular amelogenin interactions. The results from this study therefore point to a potential use of amelogenin in targeted release of pharmaceutical proteins, induced by acidification, but the charge of the entrapped protein may be critical for proper particle formation.

To test if the pH dependent aggregation properties of amelogenin could be used further to confer pH sensitivity also to other drug delivery systems, without any inherent pH responsiveness, gelatin/amelogenin microparticles were prepared using an emulsion-based method. The purpose of mixing amelogenin with gelatin was to determine if only a minor fraction of amelogenin could provide a response to pH changes in the gelatin particles, potentially giving the composite particles beneficial properties from both components. The gelatin microparticles containing amelogenin displayed a modulated structure compared to control particles without amelogenin. The difference was most pronounced at pH 7 where the amelogenin containing particles had a porous surface not observed in the control particles (Figure 4). At pH 4, a difference in the surface structure was also observed between the amelogenin and control particles, indicating that the presence of amelogenin may have effect on the particles at both acidic and neutral pH. However, the porous structure was confined to the pH 7 particles, suggesting a more pronounced effect of amelogenin on the particle surface structure at neutral pH. At pH 4, the gelatin particles appeared to lose some of their structural integrity and adapt a more flat morphology. This effect was less pronounced in the amelogenin containing particles, suggesting a stabilizing effect of amelogenin. The results clearly indicate that the amelogenin containing gelatin particles change from having a porous surface at pH 7, to having a non-porous surface when pH becomes acidic, or vice versa. The analysis carried out in this study cannot distinguish if the pores extend deeper into the particles, or if they are isolated to the surface layer. However, we propose that the change in microparticle surface porosity at pH 7 is an effect of the amelogenin solubility profile and the tendency of amelogenin to self-assemble at neutral pH. A change in amelogenin protein structure, due to changes in pH, could modulate the interaction between both amelogenin molecules and components in the gelatin, leading to a modified structure of the microparticles. Crosslinking of amelogenin with glutaraldehyde under the conditions used does not covalently attach amelogenin to gelatin (Figure 3), making it possible for amelogenin to act independently of the gelatin molecules inside the particles. The observed changes in porosity are likely to have a large effect on particle release, degradation, tissue penetration, and other pharmacologically important properties. If a porous or non-porous surface is most beneficial at a given condition depends on the application, but having the ability to change between these two states can be helpful to achieve a pH mediated delivery of substances from the particle. In addition, the results obtained in this study are supported by previous observations that an amelogenin gel, formed at pH 6.8, develops voids in response to temperature, turning the gel opaque when the temperature is changed from 4°C to 24°C [22]. The temperatures used in our study correspond with those where the voids are formed. In our study, amelogenin comprises only a small fraction (~2.5%) of the solid material in the microparticles, indicating that amelogenin confers a potent response to pH changes, even after crosslinking of the particle with glutaraldehyde. This could be a useful property when designing new drug delivery systems, or in other processes where pH responsiveness is desirable.

The amelogenin containing microparticles proved to be more resistant to trypsin degradation compared to the control particles without amelogenin (Figure 5). Since amelogenin is insoluble at pH 7, at which the proteolysis was performed, it may be a poor trypsin substrate and therefore shield the particle from degradation. Decreased trypsin sensitivity can be an advantage if an extended release in the small intestine is desired, or release further down the gastrointestinal tract. The data from this study suggest that drug delivery systems composed partly of amelogenin could have an increased tolerance towards trypsin degradation.

In our current study, we utilized native amelogenin which is insoluble between pH 6 and 7.5. By modifying the surface properties of amelogenin by for instance site-directed mutagenesis, this pH range may be modified to cover a desired pH optimum or shifting it to a wider or more narrow range. Particularly, the charged residues in the C-terminal end of amelogenin have proved to be critical in the amelogenin self-assembly [9,23]. By dissecting the influence of these residues by protein engineering methods novel amelogenins can be obtained.

## Conclusions

The results from this study clearly show that the pH dependent solubility of amelogenin makes it useful as a component in drug delivery systems, in order to create a response to changes in pH. An effect on both the entrapment/release of a target protein, and on microparticle surface porosity could be observed. Additionally, amelogenin also decreased trypsin degradation of microparticles. Human amelogenin can be made readily available by recombinant means and is suitable for use in pharmaceutical formulations since no clinical side effects have been reported over the years despite extensive use in particuarly dental surgery.

## Methods

### Preparation of recombinant amelogenin

Recombinant amelogenin (rH174) was expressed and purified from Escherichia coli BL21 (DE3) cells harboring a pET11 vector (Novagen) with an insert encoding human amelogenin (Swissprot Q99217, isoform 1, excluding the signal peptide) cloned between the NdeI and BamHI sites. The cells were cultivated at 37°C in TB-medium (100 ml in 500 ml shake flasks) and induced 4 h post inoculum with 5 mM lactose. The cells were harvested with centrifugation at 5,000 g for 10 minutes. Amelogenin was purified according to an acid/heat treatment method [17]. Briefly, the cell pellet was suspended in 3% HAc and heat treated at 80°C for 20 minutes. The insoluble fraction was removed by centrifugation at 20,000 g for 20 minutes, and the supernatant, containing soluble amelogenin, was collected. The amelogenin solution was dialyzed against 0.05% HAc, aliquoted and lyophilized. The purified amelogenin was analyzed by SDS-PAGE and the ability to form nanospheres was verified by dynamic light scattering (Zetasizer Nano, Malvern Instruments) in 100 mM HEPES, pH 8.0, at a protein concentration of 2 mg/ml.

### Molecular encapsulation with amelogenin

Use of amelogenin for pH dependent entrapment of other proteins was characterized using BSA and insulin as target molecules. For encapsulation experiments solutions of amelogenin was prepared by dissolving lyophilized amelogenin in 0.05% HAc. Concentrations of the amelogenin stock solutions used were between 10–50 mg/ml. The proteins to be encapsulated were dissolved in 0.05% HAc or in H_2_O and mixed with the amelogenin stock solution, keeping the pH below the point of amelogenin aggregation. To induce the encapsulation/aggregation the pH was adjusted to ~7.0, by slowly adding NaOH. The formed aggregates were separated from the soluble fraction by centrifugation, and the pellet was washed with 100 mM sodium phosphate buffer, pH 7.0. The pellet was re-solubilized by addition of 0.05% HAc. Samples from the different encapsulation steps were collected and analyzed with SDS-PAGE.

### Crosslinking of amelogenin and gelatin

In order to test the effects of glutaraldehyde crosslinking on amelogenin, gelatin, and mixtures thereof, the proteins were dissolved in 100 mM sodium phosphate buffer, pH 8.0, at 37°C. Glutaraldehyde was added to the samples to a final concentration of 0.5%, followed by incubation at 37°C for various periods of time (0 minutes to 2 hours). For each sample the crosslinking reaction was quenched with Tris by adding one volume of SDS-PAGE loading buffer (2% SDS, 50 mM Tris–HCl, pH 6.8, 10% glycerol, 1% mercaptoethanol, 12.5 mM EDTA, 0.02% bromophenol blue). All samples were subsequently analyzed by SDS-PAGE.

### Gelatin/amelogenin crosslinked microparticles

Composite micoparticles containing gelatin and amelogenin were prepared, in order to test the effects of amelogenin on their property and structure. Microparticles without amelogenin were also made and used as a control. The protocol used for micoparticle formation is similar to what has been previously described [4], but with some modifications. A mixture of gelatin (100 mg/ml) and recombinant amelogenin (2.5 mg/ml) solubilized in H_2_O at 40°C was transferred to sunflower oil to create an emulsion. Glutaraldehyde was added to the gelatin/amelogenin mixture to a concentration of 0.5% just before transfer to the sunflower oil. The emulsion was continuously stirred at 1 000 rpm and was incubated at 40°C for 30 minutes, followed by incubation on ice for 10 minutes. The microparticles were then isolated by adding acetone to the emulsion, which made the particles concentrate in the interphase. The oil and acetone phases were removed and the particles were transferred to a sintered glass filter and washed extensively with acetone. The particles were dried in a flow hood andsubsequently in a vaccum exsiccator.

For particle degradation studies particles were created as above, but the gelatin solution was supplemented with 4 mg/ml aniline blue.

### Physical structure of the composite microparticles

To analyze the effect of pH on the gelatin/amelogenin composite particles they were incubated for 1.5 h in either 20 mM HAc pH 4.0 or in 20 mM sodium phosphate pH 7.0. The particles were then transferred to scanning electron micropcopy (SEM) sample stubs and dried in an exsiccator, followed by drying using a lyophilizer vaccum pump. The samples were then sputter coated with gold/palladium and analyzed on an SEM instrument (Jeol JSM 6700 F NT) with 5 kV acceleration voltage.

### Proteolytic degradation of the microparticles

To determine the stability of the gelatin/amelogenin particles against proeolytic degradation the particles were incubated in 20 mM sodium phosphate pH 7.0, containing 5 μg/ml bovine pancreatic trypsin. The particles used in this experiment were supplemented with aniline blue for simple spectroscopic detection of particle degradation. The particle concentration used was 10 mg/ml and both amelogenin/gelatin particles and control particles without amelogenin were tested (four replicates/sample). The degradation was analyzed by measuring the absorbance of the supernatant at 600 nm over time on a Beckman Coulter DU 800 spectrophotometer (1 cm path length).

## Abbreviations

SEM: Scanning electron micropcopy; HAc: Acetic acid; BSA: Bovine serum albumin; Ins: Insulin; AMG: Amelogenin; SDS-PAGE: Sodium dodecylsulfate-polyacrylamide gel electrophoresis.

## Competing interests

The authors declare that they have no competing interests.

## Authors’ contributions

JSB carried out the experimental work and wrote the first draft manuscript. LB supervised the project. Both authors read and approved the final manuscript.
